# *Pleurotus albidus* Modulates Mitochondrial Metabolism Disrupted by Hyperglycaemia in EA.hy926 Endothelial Cells

**DOI:** 10.1155/2018/2859787

**Published:** 2018-06-19

**Authors:** Gabriela Gambato, Elisa Maria Pavão, Gabriela Chilanti, Roselei Claudete Fontana, Mirian Salvador, Marli Camassola

**Affiliations:** ^1^Laboratory of Enzymes and Biomass, Institute of Biotechnology, University of Caxias do Sul, Francisco Getúlio Vargas Street 1130, 95070-560 Caxias do Sul, RS, Brazil; ^2^Laboratory of Oxidative Stress and Antioxidants, Institute of Biotechnology, University of Caxias do Sul, Francisco Getúlio Vargas Street 1130, 95070-560 Caxias do Sul, RS, Brazil

## Abstract

Hyperglycaemia exacerbates the production of reactive oxygen species (ROS), contributing to the multiple complications associated with diabetes. Mitochondrial dysfunction is also known to be associated with diabetes. Therefore, the aim of this work was to study the effect of* Pleurotus albidus* extract on the mitochondrial dysfunction induced by hyperglycaemia in EA.hy926 endothelial cells. The results showed that* P. albidus* treatment prevented the increase in the activity of complex I of the electron transport chain and minimized the ROS production induced by hyperglycaemia. In addition, the extract minimized oxidative damage to lipids and proteins, caused an imbalance in the antioxidant enzyme activities of superoxide dismutase and catalase, and decreased the nitric oxide levels induced by hyperglycaemia. These data contribute to our understanding of the mitochondrial disorder induced by hyperglycaemia as well as establishing the conditions required to minimize these alterations.

## 1. Introduction

Diabetes mellitus (DM) is a metabolic disease characterized by chronic hyperglycaemia and the development of multiple complications even at the point of diagnosis [1, 2]. Prolonged or transient exposure to hyperglycaemia is associated with the generation of reactive oxygen species (ROS), which are associated with diabetic vascular complications [1]. Therefore, it is of great importance to understand the exact mechanism of generation of ROS in hyperglycaemia and to try to minimize this phenomenon. Currently available data are controversial, but ROS generation seems to originate from the NADPH oxidases (NOX) and/or from the mitochondria of the hyperglycaemic cells [3]. Excess glucose uptake by susceptible diabetic cells, such as endothelial cells, would lead to an increase in pyruvate entry into the mitochondria and an increased flux of the reduced substrates NADH (nicotinamide adenine dinucleotide) and FADH_2_ (flavin adenine dinucleotide) to the electron transport chain (ETC), which may result in dysfunction of the ETC [4]. This fact is associated with the generation, mainly in mitochondrial complex I (NADH: ubiquinone oxidoreductase) and III (Ubiquinol: cytochrome c oxidoreductase), of the superoxide anion radical (^•^O_2_^−^) [5]. An excess of ^•^O_2_^−^ leads to H_2_O_2_ formation and oxidative stress with damage to lipids, proteins and DNA, cell dysfunction, and apoptosis, which are important factors in the pathophysiology of diabetes and its complications [3, 6, 7]. Therefore, in recent years several studies have investigated the effect of phenolic compounds that specifically reduce ROS. Tea polyphenols, high-polyphenol chocolate, and citrus fruit [1] have been studied both in* in vitro* and* in vivo* models of hyperglycaemia. Although in all cases the compounds had positive effects on minimizing cell dysfunction the exact mechanism of the biological effects remains to be understood. The genus* Pleurotus* contains a large number of edible mushroom species that have economic and ecological value and medicinal properties [8, 9]. This genus is rich in phenolic compounds, which are natural metabolites that are widely distributed in fruits and vegetables that are part of the human diet [10].* Pleurotus albidus* is cultivated in many parts of the world since it is able to grow under different climatic conditions on cheap, readily available waste materials [8]. It has recently been demonstrated that some phenolic compounds are able to modulate the activity of the ETC and its associated mitochondrial ROS pathways [1].

Therefore, the aim of this study was first, to cultivate the mushroom* P. albidus* and to determine its macronutrient, polyphenol, and ergothioneine content, as well as its antioxidant activity. Second, the influence of hyperglycaemia and the effect of* P. albidus* extract on mitochondrial dysfunction and redox metabolism were assayed in hyperglycaemic EA.hy926 endothelial cells.

## 2. Material and Methods

### 2.1. Chemicals and Reagents

Dulbecco's modified eagle medium (DMEM), fetal bovine serum (FBS), trypsin-EDTA, penicillin-streptomycin, and trypan blue were purchased from Gibco BRL (Grand Island, NY, USA). The Cell-Titer-Glo® kit was from Promega (Madison, WI). All other reagents and solvents were obtained from Sigma (St. Louis, MO, USA). All of the chemicals were of analytical grade.

### 2.2. Fungal Collection

The fungal strain used in this work was* P. albidus* 88F. This wild strain was collected in São Francisco de Paula city, Rio Grande do Sul, Brazil, with permission from the Brazilian Institute of Environment and Renewable Natural Resources (IBAMA) “special access authorization and shipment of samples of genetic heritage components” number 02001.007656/2012-11. The mycelia from the isolate have been deposited in the collection of microorganisms of the Enzymes and Biomass Laboratory, Institute of Biotechnology, University of Caxias do Sul, Rio Grande do Sul, Brazil. The macrofungi collected after isolation in artificial medium were dried and deposited in the Mycological Collection at the Herbarium of the University of Caxias do Sul.

### 2.3. *Pleurotus albidus* Cultivation and Extract Preparation

Solid-state fermentation was carried out in polypropylene bags containing 1 kg of substrate (94% (w/w)* Pinus* spp. sawdust, 5% (w/w) wheat bran, and 1% (w/w) calcium carbonate). The mushrooms were harvested daily, whenever fruiting bodies formed [8]. The* P. albidus* extract was prepared according to [8]. For this, 10 g of dry powdered mushroom and 100 mL of ethanol 70% were placed in a reflux system for 30 min at 100°C. Then the mixture was filtered under vacuum and the ethanol soluble fraction was evaporated under reduced pressure in a rotary evaporator (model 803, Fisatom, Brazil). The ethanol-insoluble residue was reextracted with 100 mL of water for another hour in the same system. The extracts were pooled and lyophilized (Benchtop Freeze Dry System, Labconco) until the* P. albidus* dry extract (PleExt) was obtained.

### 2.4. Determination of* Pleurotus albidus* Macronutrients

The cultivated mushrooms were dried at 50°C in air circulation (Fun Kitchen® food dehydrator - 5100968). They were ground into powder with a knife mill (coffee grinder Cadence®). The carbohydrate, lipid, protein, total fibre, ash, and moisture content of dry* P. albidus* were determined using Association of Official Analytical Chemists methods.

### 2.5. PleExt Total Phenolic and Flavonoid Content

The Folin-Ciocalteu method was used to determine the PleExt phenolic content. Briefly, 200 *µ*L of PleExt 2.5 mg/mL was mixed with 800 *µ*L of 7.5% sodium carbonate (w/v) and 1000 *µ*L of Folin-Ciocalteu reagent. The mixture was incubated at room temperature for 30 min then the absorbance was read at 765 nm in a spectrophotometer (UV-1700 spectrophotometer, Shimadzu, Kyoto, Japan). The results were expressed as milligrams gallic acid equivalents (mg of GA/g PleExt). Total flavonoid content was determined using the colorimetric method described previously by Woldegiorgis* et al.* (2014). Briefly, an aliquot of 250 *µ*L of the PleExt 2.5 mg/mL or quercetin standard solutions was mixed with 1.25 mL of distilled water in a test tube, followed by adding 75 *µ*L of a NaNO_2_ solution (5% (w/v) in water). After 6 min, 150 *µ*L of the 10% AlCl_3_.6H_2_O solution was incubated for 5 min and then added to 0.5 mL of 1 mol/L NaOH. The mixture was made up to 2.5 mL with distilled water and mixed well. The absorbance was measured immediately against the blank at 425 nm. The results were calculated and expressed as milligrams of quercetin equivalents (mg of QE/g PleExt).

### 2.6. Ergothioneine Quantification

The method used to quantify ergothioneine used 0.5 g of PleExt was diluted in 12 mL of water and then filtered through a 0.22 *µ*m membrane. The analysis was carried out at ambient temperature using an HPLC (Shimadzu, Kyoto, Japan) and UV-VIS detector equipped with a 20 *µ*L injection volume. Separation was carried out on C18 columns (Discovery®) (150 × 4.6 mm and 5 *μ*m particle size). The isocratic mobile phase was 50 mmol/L sodium phosphate in water with 3% acetonitrile and 0.1% triethylamine adjusted to a pH of 7.3 with a flow rate of 0.4 mL per minute. Ergothioneine was quantified by monitoring absorbance at 254 nm and comparing the peak area of the sample to peak areas obtained from different concentrations of the standard. Data was expressed as milligrams of ergothioneine per gram of PleExt (mg/g PleExt). The analysis was performed in triplicate.

### 2.7. *In Vitro* Antioxidant Activity

Free radical scavenging activity was determined using 2,2-diphenyl-1-picrylhydrazyl (DPPH^•^) and ABTS^•+^ (2,2-azino-bis(3-ethylbenzthiazoline-6-sulfonic acid) methods. A 200 *µ*L volume of extract solution (2.5, 5, 10, and 20 mg/mL) was added to 800 *µ*L of Tris-HCL buffer (100 mmol/L pH 7.4) and then mixed with 1 mL of DPPH^•^ (500 *µ*mol/L dissolved in ethanol). The mixture was kept in the dark at room temperature for 20 minutes. The absorbance was read at 517 nm (UV-1700 spectrophotometer, Shimadzu, Kyoto, Japan). The result was expressed as the half-maximal inhibitory concentration (IC_50%_) in mg/mL. The capacity of the extract to reduce the ABTS^•+^ radical cation was determined following a previously published method with some modification (Re* et al*., 1999). For this 30 *µ*L of each dilution of the PleExt extract was reacted with 3 mL of the ABTS^•+^ solution (diluted with ethanol to an absorbance of 0.70 ± 0.02). The absorbance was measured after 6 minutes at 734 nm. The result was expressed as the half-maximal inhibitory concentration (IC_50%_) in mg/mL.

### 2.8. Hyperglycaemic Cell Culture

The EA.hy926 human vascular endothelial cell line was purchased from Rio de Janeiro Cell Bank and maintained in DMEM high glucose containing 10% FSB, 100 U/mL penicillin, and 100 mg/mL streptomycin at 37°C under an atmosphere of 5% CO_2_. The induction of hyperglycaemia was carried out as previously described by [11]. An additional 35 mmol/L glucose was added to the medium for the hyperglycaemic groups (HG). DMEM high (25 mmol/L) glucose medium was used for the control group (NG).

### 2.9. Experimental Design

Mitochondrial dysfunction and the redox metabolism were evaluated by treating NG and HG cells with 0.1 *µ*g/mL, 0.5 *µ*g/mL, and 1.0 *µ*g/mL PleExt in a cotreatment assay. After 5 days of treatment, the cells were washed with cold phosphate-buffered saline, resuspended in 20 mmol/L hypotonic potassium phosphate buffer (pH 7.5), and the cell lysate was snap-frozen in liquid nitrogen and thawed at 37°C three times [12]. The sample was divided into aliquots and stored at -80°C until required for the assays.

### 2.10. Mitochondrial Respiratory Chain Assessment

The assessment of complex I, complex II, and complex III activity was performed using a single-wavelength, temperature-controlled spectrophotometer (UV-1700 spectrophotometer, Shimadzu, Kyoto, Japan) at 37°C [12]. For complex I, the sample was equilibrated for 2 min in reaction buffer (0.5 mol/L potassium phosphate buffer pH 7.5, 50 mg/mL acid-free bovine serum albumin (BSA), and 10 mmol/L of KCN and NADH) and then the reaction was started by adding 6 *µ*l of ubiquinone (10 mmol/L). The decrease in absorbance over 2 min was read at 340 nm (UV-1700 spectrophotometer, Shimadzu, Kyoto, Japan). The result was expressed as complex I enzyme activity (nmol min^-1 ^mg^−1^ protein). To assay complex II activity, the sample was incubated for 10 min in reaction buffer (0.5 mmol/L potassium phosphate buffer pH 7.5, 50 mg/mL of fatty acid-free BSA, 10 mmol/L KCN, 400 mmol/L succinate, and 0.015% (w/v) 2.6-dichlorophenolindophenol sodium salt hydrate). The decrease in absorbance over 3 min was read at 600 nm after addition of 4 *µ*l of 12.5 mmol/L decylubiquinone. The result was expressed as complex II enzyme activity (nmol min^−1^ mg^−1^ protein). For complex III, the sample was incubated for 2 min in reaction buffer (0.5 mol/L potassium phosphate buffer pH 7.5, 10 mmol/L of KCN, and 5 mmol/L ethylene diamine tetra acetic acid disodium salt dihydrate pH 7.5 and Tween-20 2.5% (v/v)), then the reaction was started by adding 10 *µ*l of decylubiquinol (10 mmol/L). The increase in absorbance over 2 min was read at 550 nm. The result was expressed as complex III enzyme activity (nmol min^−1^ mg^−1^ protein). ATP levels were measured using the Cell-Titer-Glo® Assay Kit, according to the manufacturer's instructions. The result was expressed as a % of the normal glucose group.

### 2.11. Cellular Viability

The viability of cells was determined by conventional light microscopy using trypan blue staining. EA.hy926 cells were plated on six-well-plates. After 5 days of treatment, the cells were detached and exposed to the trypan blue reagent (0.4%) for 10 min. The percentage viability was calculated as the number of unstained cells/total number of cells × 100. The results represent the average of three independent experiments performed in triplicate.

### 2.12. Intracellular ROS Generation

Generation of intracellular ROS was assessed using the oxidative-sensitive fluorescent probe 2′-7′-dichlorofluorescein diacetate (DCFH-DA). EA.hy926 cells in different treatment conditions were loaded with 10 *μ*mol/L DCFH-DA in culture media. DCFH-DA is hydrolyzed by intracellular esterases to dichlorofluorescein (DCFH), which is trapped within the cell. This nonfluorescent molecule is then oxidized to fluorescent dichlorofluorescein (DCF) by the action of cellular oxidants. Following exposure to the DCFH-DA, the cells were washed three times with fresh phosphate-buffered saline. Then the cells were harvested according to a previously published method [13]. Fluorescence was measured using a plate reader (SpectraMax® Plus Microplate Reader) at excitation and emission wavelengths of 485 nm and 520 nm, respectively.

### 2.13. Oxidative Lipid and Protein Content

Oxidative lipid was monitored by the formation of thiobarbituric acid reactive substances (TBARS) during an acid-heating reaction according to [14]. Hydrolyzed 1,1,3,3-tetramethoxypropane (TMP) was used as the standard, and the results were expressed as nmol TMP/mg protein. Oxidative damage to proteins was measured based on the reaction of protein carbonyl groups with 2.4-dinitrophenylhydrazine (DNPH). The absorbance was read (UV-1700 spectrophotometer, Shimadzu, Kyoto, Japan) at 365 nm, and results were expressed as nmol DNPH/mg protein.

### 2.14. Superoxide Dismutase and Catalase Activities

Superoxide dismutase (SOD) activity was measured by the rate of inhibition of self-catalytic adrenochrome formation at 480 nm, in a reaction medium containing 1 mmol/L adrenaline (pH 2.0) and 50 mmol/L glycine (pH 10.2) at 30°C for 3 min. The results were expressed as U/mg protein. One SOD unit (U) is defined as the amount of enzyme that inhibits the rate of adrenochrome formation by 50% [15]. Catalase (CAT) activity was measured by the rate of H_2_O_2_ decomposition at 30°C for 1 min in 240 nm. The results were expressed as U/mg protein. One CAT unit is defined as the amount of enzyme that decomposes 1 mmol of H_2_O_2_ in 1 min at pH 7.4.

### 2.15. Nitric Oxide Levels

For the evaluation of nitric oxide (NO) levels, 100 *μ*L of cell lysate was reacted with an equal volume of Griess reagent (0.1% naphthylethylenediamine and 1% sulfanilamide in 5% H_3_PO_4_) for 10 min at room temperature, and the absorbance was read at 550 nm (UV-1700 spectrophotometer, Shimadzu, Kyoto, Japan). Sodium nitroprusside was used as the standard. The results were expressed as nmol of nitrite/mg protein.

### 2.16. Protein Concentration

In order to determine the protein level the Lowry method was performed in cell lysate. BSA was used as the standard.

### 2.17. Statistical Analysis

The results were expressed as the mean ± standard deviation (SD) of at least three independent experiments. The software SPSS 21.0 (SPSS Inc., Chicago, IL) was used. The results were determined to be parametric using the Kolmogorov-Smirnoff test. Statistical significance was evaluated using one-way analysis of variance (ANOVA), followed by Tukey's post hoc test. The relationships between the variables were assessed using Pearson's correlation coefficient. Statistical significance was determined at* p* < 0.05.

## 3. Results

**Figure 1:**
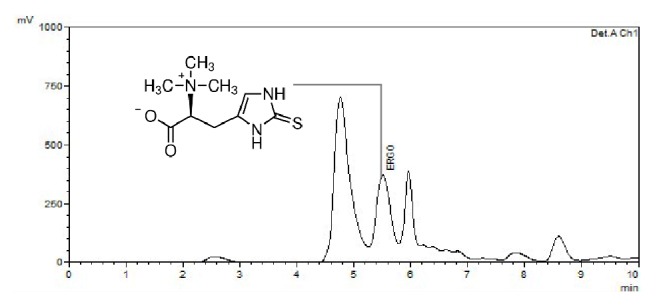
HPLC UV-VIS chromatogram showing the presence of ergothioneine in* Pleurotus albidus* extract (PleExt). Ergothioneine was identified by comparing the peak of the sample to the peak of the standard.

Dietary fibre (42.20 ± 0.10 g/100 g) and protein (21.82 ± 0.12 g/100 g) were the main macronutrients in* P. albidus* dry mushroom (Table 1).* P. albidus* also contained carbohydrate 17.63 ± 0.50 g /100 g, ash (5.87 ± 0.09 g /100 g) and low levels of fat (3.72 ± 0.06 g/100 g). The extract obtained (PleExt) was an important source of phenolic compounds (18.57 ± 0.26 mg GAE/g), about 20% of them represented by flavonoids (4.71 ± 0.12 mg QE/g). The extract showed important antioxidant activity in both the DPPH^•^ and ABTS^•+^ assays. The IC_50_ in the DPPH^•^ and ABTS^•+^ assays was 21.62 ± 1.02 mg/mL and 12.47 ± 0.16 mg/mL PleExt, respectively. We observed a positive correlation between* in vitro* antioxidant activity assayed by DPPH^•^ (r = 0.969,* p* < 0.001) and ABTS^•+^ (r = 0.993,* p* < 0.001) and the phenolic content of the mushroom. The PleExt contained 2.13 ± 0.01 mg/g of ergothioneine identified by HPLC UV-VIS (Figure 1).

**Figure 2:**
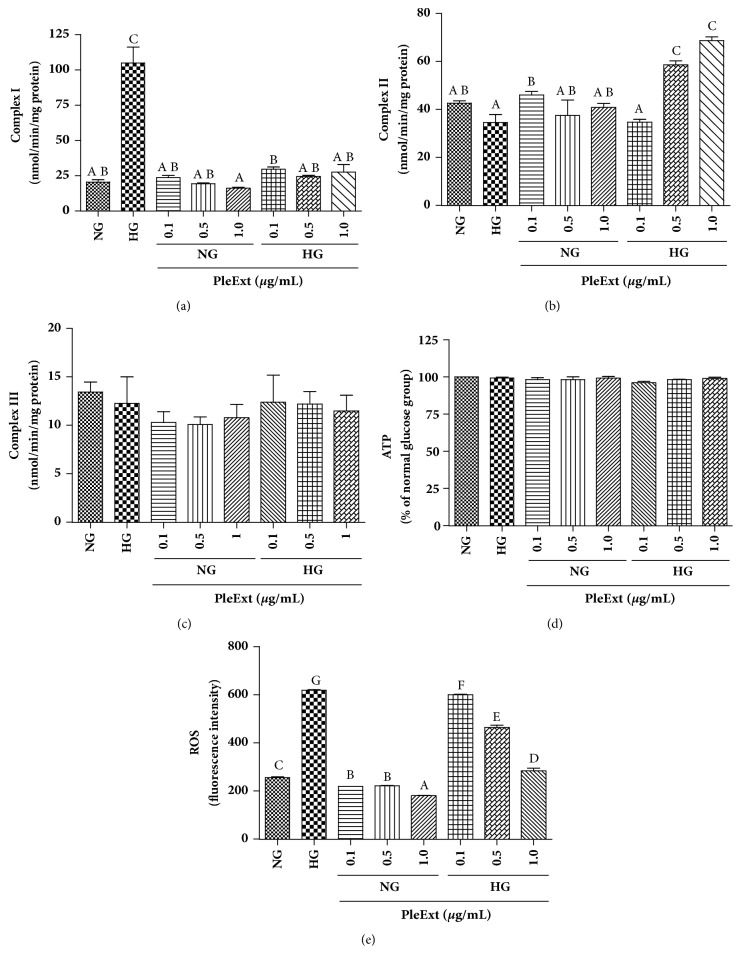
Mitochondrial respiratory chain assessment and intracellular reactive oxygen species (ROS) generation by endothelial cells treated with normal glucose (NG) or high glucose (HG) plus* P. albidus extract* (PleExt). (a) Complex I activity. (b) Complex II activity. (c) Complex III activity. (d) ATP levels. (e) Intracellular ROS generation. Results are expressed as mean ± SD of at least three independent experiments. Different letters indicate significantly different values among the treatments according to the analysis of variance (ANOVA) and Tukey's post hoc test. Statistical significance was determined at* p* < 0.05.

**Figure 3:**
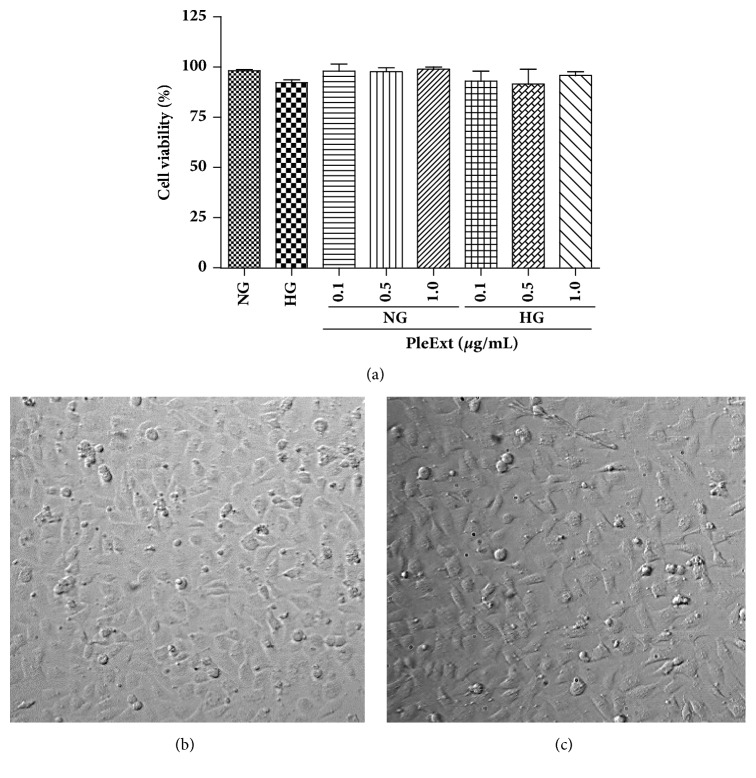
Effects of normal glucose (NG) or high glucose (HG) plus* P. albidus extract* (PleExt) on EA.hy926 endothelial cell viability. (a) Cell viability by trypan blue assay. Cell morphology under light microscopy (100x) at (b) normal glucose and (c) high glucose levels.

Figure 2(a) shows that in hyperglycaemic conditions the EA.hy926 endothelial cells showed higher complex I activity and unchanged activity of complex II (Figure 2(b)) and III (Figure 2(c)) activity relative to control conditions. ATP production (Figure 2(d)) and cell viability (Figure 3) did not change in hyperglycaemic conditions. PleExt treatments prevented the increase in complex I activity and minimized the ROS production (Figure 2(e)) induced by hyperglycaemic conditions. Complex I activity showed a positive correlation (r = 0.6741,* p* < 0.01) with the levels of ROS in endothelial cells. Complex II activity was higher in hyperglycaemic cells treated with 0.5 and 1.0 *µ*g/mL of PleExt when compared to normal and high glucose cells (Figure 2(b)).

The oxidative lipid and protein damage and the modulation of enzymatic antioxidant systems and NO levels are described in Table 2. In high glucose cells, we observed an increase in lipid and protein oxidative damage and a depletion of the activities of the antioxidant enzymes SOD and CAT as well as of the NO levels. PleExt treatments minimized the oxidative damage to both lipids and proteins, mainly when used at the higher concentrations. Positive correlations between ROS levels and lipid damage (r = 0.905,* p* < 0.01) and between ROS levels and carbonyl protein (r = 0.942,* p* < 0.01) were observed. The imbalance in the activities of SOD and CAT were minimized after treatment with PleExt. All concentrations of PleExt assayed were able to restore the decline in NO levels induced by hyperglycaemic conditions. The treatment of normal glucose cells with only PleExt had no effect on any of the evaluated markers.

## 4. Discussion

Mitochondria play a key role in the management of several cellular functions, including stress responses and physiological metabolism. They are the principal source of excessive ROS formation in cells, which has a negative impact on electron transport. It is already known that hyperglycaemia is an adverse condition that contributes to a breakdown in the bioenergetics functions of mitochondria [16]. It has been reported that cells in high levels of glucose show impaired electron transport chain complex activities. However, the exact alterations of ETC are controversial. In this study hyperglycaemic cells showed around fourfold higher complex I activity than normal cells, probably because of the high flow of NADH substrate in the ETC. This effect was accompanied by an increase in ROS, lipid and protein oxidative damage, and a decrease in SOD and CAT activities. The mitochondrial ETC complex I is a great producer of ^−^O_2_^•^ [17] and this ROS is dismutated by the action of the antioxidant enzyme SOD, generating hydrogen peroxide (H_2_O_2_). Hydrogen peroxide serves as a substrate for the action of the cytosolic antioxidant enzyme CAT, which generates water and oxygen from the catalysis of H_2_O_2_ [5]. The depletion of enzyme activities is probably a response to the high levels of ROS produced due to complex I dysfunction. As antioxidant defenses are depleted, more ROS are produced, which can damage the ETC structure and cause oxidative damage to lipids and proteins [14]. Therefore, the initial complex I inhibition becomes the feedback for augmenting its own inhibition [18].* P. albidus* treatment prevented the increase in complex I activity, as well as the increase in ROS and lipid and protein oxidative damage induced by hyperglycaemic conditions. Besides, the extract modulated SOD and CAT activities, thus maintaining the antioxidant defenses. Curiously, treatment of hyperglycaemic cells with 0.5 and 1.0 mg/mL of PleExt led to an increase in complex II activity; more studies are needed to understand this data.

Endothelium plays an important role in the production of NO, a vasodilator molecule. Through the endothelial nitric oxide synthase (eNOS), endothelial cells can generate NO from its precursor L-arginine with the cofactor tetrahydrobiopterin [19]. Studies have shown that endothelial hyperglycaemic conditions can uncouple eNOS and impair NO production [19].* P. albidus* extract was able to restore the decline in NO levels induced by hyperglycaemic conditions. This finding is important because viable endothelial cells in disrupted NO levels can signal to inflammatory state by the vasoconstriction and leukocyte adherence [20]. This imbalance can contribute to the pathophysiology of several cardiovascular diseases such as hypertension and atherosclerosis [21].

It has already been shown that ergothioneine can reduce inflammation in human endothelial cells through the expression of eNOS. Mammalian tissues have a highly specific transporter for ergothioneine, named OCTN1, which implies a possible biological role for this molecule in cells [22]. The major source of ergothioneine is mushrooms, such as* Pleurotus ostreatus* and* Lentinus edodes* [23]. Ergothioneine presents antioxidant and anti-inflammatory activity in endothelial cells [24]. The ergothioneine content of PleExt (2.13 ± 0.01 mg/g) probably contributes to its mitochondrial biological activity, but more studies about ergothioneine and the anti-inflammatory response are required to determine the exact relation between them.

This study reports the main constituents of* P. albidus* for the first time, showing that the mushroom is an important source of proteins with low levels of fats. Besides, PleExt showed important antioxidant activity and a high level of polyphenols, mainly flavonoids, which were found at levels higher than those observed for another mushroom of the same genus,* P. eryngii* [25]. Interest in phenolic compounds has grown during recent decades due to the recognition of their antioxidant properties and their probable role in the prevention of a number of pathologies associated with oxidative stress [26]. One of the limitations of our work is the lack of data about which phenolic compounds are present in PleExt. This information would be useful to understand which molecules are involved in the biological effect observed. On the other hand, it is already known that mushrooms of the same genus, such* P. ostreatus*, contain gallic and chlorogenic acids and the flavonoids naringin and hesperetin [27]. Quercetin, rutin, hesperidin, and myricetin flavonoids have already been reported in* P. tuberregium* [28]. These molecules are known to provide health benefits and antioxidant effects [19], and the use of more than one phenolic compound could give rise to synergism, allowing distinct targeting of dysfunctional biochemical pathways. Besides the phenolic compounds, PleExt also contained ergothioneine, a molecule that is biosynthesized in some bacteria and fungi but not in animals [29].

**Figure 4:**
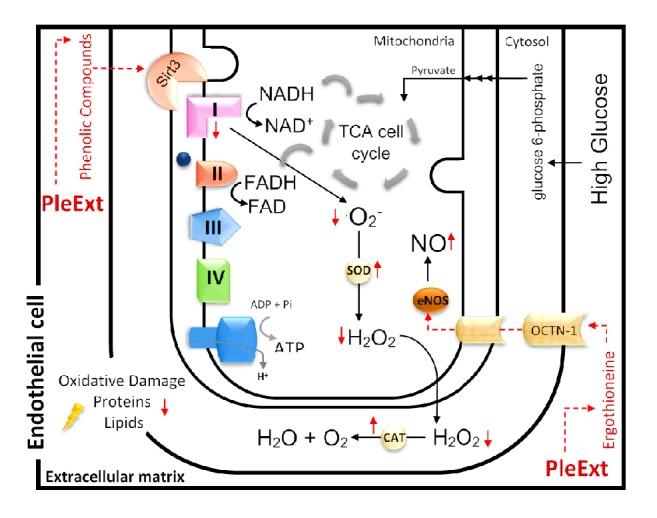
Hypothetical mechanisms of action of* Pleurotus albidus* extract (PleExt) in EA.hy926 under high glucose. PleExt can activate Sirt 3, which helps normalize complex I activity and thus reduce ^•^O_2_^−^ production. Therefore, the oxidative damage to lipids and proteins was also diminished. Ergothioneine, a key substrate for OCTN1, was found in PleExt and can be transported to intracellular compartments and modulate eNOS activity to normalize NO production.

The mechanism by which PleExt modulates the activity of complex I is not known. However, studies have already shown that catechin, resveratrol, and quercetin are capable of directly or indirectly increasing the levels of proteins called sirtuins [26]. These classes of molecules are mainly protein deacetylases that are involved in diverse cellular processes and pathways, and they present different cell localizations and functions. Seven sirtuins have already been described in mammals, named SIRT1 to SIRT7 [30]. SIRT3 is the major mitochondrial deacetylase, and it regulates complex I activity, keeping the electron chain functional. It has already been shown that hyperglycaemia is associated with the reduced activity of all SIRT (1 to 7) in mammalian cells [30]. Although the data obtained in our study do not provide definitive evidence, it is possible that PleExt targets SIRT3, maintaining normal complex I activity and therefore decreasing ROS production and oxidative damage to lipids and proteins (Figure 4). It has previously been reported that epicatechin-rich cocoa increased the expression of SIRT3 in the skeletal muscle of patients with type II diabetes and heart failure [31]. Other studies should be done to clarify this hypothesis and to provide perspectives for the use of sirtuins as new targets to treat hyperglycaemia.

This study demonstrated that PleExt of* P. albidus *(88F), a source of phenolic compounds and ergothioneine, prevents the decline in the activity of complex I of the ETC and minimizes the oxidative damage induced by hyperglycaemia. Although extrapolation of the results of cell culture studies to human clinical situations is uncertain, this is an important finding for possible therapeutic use and the development of new therapeutic agents that could reduce the adverse effects of hyperglycaemic conditions.

## Figures and Tables

**Table 1:** Main macronutrients in *Pleurotus albidus* dry mushroom and chemical compounds of *P. albidus* extract (PleExt).

Compound*∗*	Content
Energy (kcal/100 g)	191.27 ± 0.15
Carbohydrate (g/100 g)	17.63 ± 0.50
Dietary fibre (g/100 g)	42.20 ± 0.10
Fat (g/100 g)	3.72 ± 0.06
Moisture (g/100 g)	8.76 ± 0.03
Proteins (g/100 g)	21.82 ± 0.12
Ash (g/100 g)	5.87 ± 0.09
Phenolics (mg GAE/g PleExt)	18.57 ± 0.26
Flavonoids (mg QE/g PleExt)	4.71 ± 0.12
Ergothioneine (mg/g PleExt)	2.13 ± 0.01

*∗*Results are expressed as mean ± SD of at least three independent experiments. Phenolic content is expressed as milligrams of gallic acid equivalents (mg of GA/g PleExt) and flavonoid content is expressed as milligrams of quercetin equivalents (mg of GA/g PleExt).

**Table 2:** Modulation of oxidative stress markers by *Pleurotus albidus* extract (PleExt) in EA.hy926 endothelial cell under hyperglycaemia.

Treatment	TBARS (nmol TMP/mg protein)	Carbonyl protein (nmol DNPH/mg protein)	SOD (U/mg protein)	CAT (U/mg protein)	NO (nmol nitrite/mg protein)
NG	0.18 ± 0.01^a*∗*^	0.83 ± 0.03^a^	179.33 ± 0.57^d^	19.52 ± 2.77^bc^	0.37 ± 0.05^b^
HG	0.35 ± 0.01^b^	5.84 ± 0.30^b^	134.43 ± 2.89^b^	11.54 ± 1.45^a^	0.11 ± 0.01^a^
NG + PleExt 0.1 *µ*g/mL	0.15 ± 0.01^a^	0.63 ± 0.03^a^	165.57 ± 5.09^cd^	21.47 ± 1.43^c^	0.34 ± 0.08^b^
NG + PleExt 0.5 *µ*g/mL	0.12 ± 0.02^a^	0.50 ± 0.03^a^	172.20 ± 4.24^d^	21.14 ± 4.21^c^	0.35 ± 0.07^b^
NG + PleExt 1.0 *µ*g/mL	0.14 ± 0.03^a^	0.40 ± 0.06^a^	170.12 ± 1.55^d^	20.56 ± 2.94^bc^	0.38 ± 0.09^b^
HG + PleExt 0.1 *µ*g/mL	0.31 ± 0.06^b^	4.23 ± 0.50^c^	123.71 ± 1.15^a^	13.39 ± 1.02^ab^	0.36 ± 0.01^b^
HG + PleExt 0.5 *µ*g/mL	0.26 ± 0.03^c^	2.08 ± 0.21^d^	138.28 ± 0.14^b^	15.62 ± 2.87^ab^	0.42 ± 0.02^b^
HG + PleExt 1.0 *µ*g/mL	0.17 ± 0.02^a^	1.88 ± 0.01^d^	160.48 ± 0.63^c^	21.09 ± 2.04^c^	0.35 ± 0.08^b^

Content of oxidative stress markers in normal glucose (NG) or high glucose (HG) plus *P. albidus* extract (PleExt) after 5 days treatment. ^*∗*^Results are expressed as mean ± SD of at least three independent experiments. Different letters indicate significantly different values among treatments according to the analysis of variance (ANOVA) and Tukey's post hoc test. Statistical significance was determined at *p < *0.05.

## Data Availability

The data used to support the findings of this study are available from the corresponding author upon request.
